# Supplementary material for: Helicobacter pylori Eradication Does Not Adversely Affect the Clinical Course of Gastric Cancer: A Multicenter Study on Screening Endoscopic Examination in Japan
Source: Cancers (Basel). 2024 Feb 9;16(4):733. doi: 10.3390/cancers16040733 (PMC10887210; doi:10.3390/cancers16040733)
Supplement: Supplementary file 1 [file cancers-16-00733-s001.zip › cancers-2836718-supplementary.pdf]

Table S1. Baseline characteristics of the study patients before and after propensity score matching, when defining *HP*-eradicated GCs were defined as those who were diagnosed at least two years after successful eradication.

	**Before propensity score matching**				**After propensity score matching**			
	***HP*-eradicated (*n* = 83)**	***HP*-positive (*n* = 97)**	***p* value**	**ASD**	***HP*-eradicated (*n* = 72)**	***HP*-positive (*n* = 72)**	***p* value**	**ASD**
Male sex, *n* (%)	72 (86.7)	84 (86.6)	1.00	<0.01	64 (88.9)	66 (91.7)	0.78	0.09
Age, years, median (IQR)	65 (61–70)	64 (59–70)	0.33	0.14	65 (61–70.3)	66 (59.8–70)	0.66	0.06
Metachronous cancer, *n* (%)	8 (9.6)	7 (7.2)	0.60	0.09	7 (9.7)	6 (8.3)	1.00	0.05
Smoking status, *n* (%)								
Current smoker	23 (27.7)	28 (28.9)	0.94	0.05	21 (29.2)	25 (34.7)	0.74	0.13
Past smoker	14 (16.9)	17 (17.5)			12 (16.7)	10 (13.9)		
Never smoker	44 (53.0)	48 (49.5)			39 (54.2)	37 (51.4)		
Unknown	2 (2.4)	4 (4.1)			0 (0.0)	0 (0.0)		
Drinking status, *n* (%)								
Current drinker	62 (74.7)	68 (70.1)	0.67	0.15	58 (80.6)	58 (80.6)	1.00	<0.01
Past drinker	1 (1.2)	3 (3.1)			0 (0.0)	0 (0.0)		
Never drinker	16 (19.3)	21 (21.6)			14 (19.4)	14 (19.4)		
Unknown	4 (4.8)	5 (5.2)			0 (0.0)	0 (0.0)		
Longitudinal location, *n* (%)								
Upper third	17 (20.5)	15 (15.5)	0.44	0.13	16 (22.2)	13 (18.1)	0.68	0.10
Middle or lower third	66 (79.5)	82 (84.5)			56 (77.8)	59 (81.9)		
Macroscopic type, *n* (%)								
Elevated type	14 (16.9)	24 (24.7)	0.20	0.20	13 (18.1)	18 (25.0)	0.42	0.17

Depressed type	69 (83.1)	73 (75.3)			59 (81.9)	54 (75.0)		
Tumor size, *n* (%)								
<20 mm	58 (69.9)	55 (56.7)	0.09	0.28	50 (69.4)	38 (52.8)	0.06	0.35
≥20 mm	25 (30.1)	42 (43.3)			22 (30.6)	34 (47.2)		
Histological type, *n* (%)								
Differentiated type	69 (83.1)	83 (85.6)	0.68	0.07	60 (83.3)	62 (86.1)	0.82	0.08
Undifferentiated type	14 (16.9)	14 (14.4)			12 (16.7)	10 (13.9)		
Interval between previous and diagnostic endoscopic examination, *n* (%)								
≤1 year	54 (65.1)	30 (30.9)	<0.01	0.93	48 (66.7)	22 (30.6)	<0.01	0.96
>1 year, ≤ 2 years	17 (20.5)	15 (15.5)			15 (20.8)	13 (18.1)		
>2 years or never	12 (14.5)	52 (53.6)			9 (12.5)	37 (51.4)		
Depth of tumor invasion, *n* (%)								
T1a	58 (69.9)	72 (74.2)	0.66	0.19	49 (68.1)	49 (68.1)	0.76	0.19
T1b1	7 (8.4)	5 (5.2)			6 (8.3)	5 (6.9)		
T1b2	14 (16.9)	13 (13.4)			14 (19.4)	12 (16.7)		
T2 or deeper	4 (4.8)	7 (7.2)			3 (4.2)	6 (8.3)		

ASD absolute standardized difference; *HP Helicobacter pylori*; *IQR* interquartile range.

Table S2. Risk of T1b–T4-GC associated with HP eradication, when defining *HP*-eradicated GCs were defined as those who were diagnosed as at least two years after successful eradication.

		**Adjusted OR [a]**	**95% CI**	***p* value**
Longitudinal location	Upper third	2.96	1.10–7.93	0.03
	Middle or lower third	reference		
Macroscopic type	Depressed type	2.07	0.68–6.34	0.20
	Elevated type	reference		
Tumor size	≥20 mm	6.33	2.69–14.90	<0.01
Histological type	Undifferentiated type	2.03	0.70–5.86	0.19
	Differentiated type	reference		
Interval between previous and diagnostic endoscopic examination	≤1 year	reference		
	>1 year, ≤2 years	1.39	0.43–4.45	0.58
	>2 years or never	4.35	1.51–12.50	<0.01
HP infection status	*HP*-eradicated	2.43	0.91–6.49	0.08
	HP-positive	reference		

CI confidence interval; *GC* gastric cancer; *HP Helicobacter pylori*; *OR* odds ratio; [a] Adjusted by longitudinal location, macroscopic type, tumor size, histological type, interval between previous and diagnostic endoscopic examination, and *HP* infection status.

Table S3. Risk of T1b2–T4-GC associated with HP eradication, when defining *HP*-eradicated GCs were defined as those who were diagnosed as at least two years after successful eradication.

		Adjusted OR [a]	95% CI	*p* value
Longitudinal location	Upper third	3.93	1.32–11.70	0.01
	Middle or lower third	reference		
Macroscopic type	Depressed type	4.15	0.98–17.50	0.053
	Elevated type	reference		
Tumor size	≥20 mm	9.01	3.25–25.00	<0.01
Histological type	Undifferentiated type	2.17	0.70–6.76	0.18
	Differentiated type	reference		
Interval between previous and diagnostic endoscopic examination	≤1 year	reference		
	>1 year, ≤2 years	2.50	0.66–9.49	0.18
	>2 years or never	6.28	1.83–21.60	<0.01
HP infection status	*HP*-eradicated	2.76	0.89–8.61	0.08
	HP-positive	reference		

CI confidence interval; *GC* gastric cancer; *HP Helicobacter pylori*; *OR* odds ratio; [a] Adjusted by longitudinal location, macroscopic type, tumor size, histological type, interval between previous and diagnostic endoscopic examination, and *HP* infection status.

Table S4. Clinical characteristics of the study patients with pT1 and pT2 or deeper GC.

	**T1** (***n*** **= 215)**	**T2 or deeper** (***n*** **= 16)**	***p*** **value**
Male sex, *n* (%)	183 (85.1)	16 (100.0)	0.14
Age, years, mean (SD)	65 (60–70)	65 (59–68.3)	0.57
Metachronous cancer, *n* (%)	18 (8.4)	0 (0.0)	0.62
Smoking status, *n* (%)			
Current smoker	55 (25.6)	8 (50.0)	0.15
Past smoker	42 (19.5)	2 (12.5)	
Never smoker	109 (50.7)	6 (37.5)	
Unknown	9 (4.2)	0 (0.0)	
Drinking status, *n* (%)			
Current drinker	147 (68.4)	13 (81.2)	0.87
Past drinker	8 (3.7)	0 (0.0)	
Never drinker	48 (22.3)	3 (18.8)	
Unknown	12 (5.6)	0 (0.0)	
Longitudinal location, *n* (%)			
Upper third	37 (82.8)	6 (37.5)	0.09
Middle or lower third	178 (82.8)	10 (62.5)	
Macroscopic type, *n* (%)			
Elevated type	45 (20.9)	1 (6.2)	0.21
Depressed type	170 (79.1)	15 (93.8)	
Tumor size, *n* (%)			

<20 mm	141 (65.6)	4 (25.0)	<0.01
≥20 mm	74 (34.4)	12 (75.0)	
Histological type, *n* (%)			
Differentiated type	187 (87.0)	8 (50.0)	<0.01
Undifferentiated type	28 (13.0)	8 (50.0)	
Duration between previous and diagnostic endoscopic examination, *n* (%)			
≤1 year	102 (47.4)	6 (37.5)	0.66
>1 year, ≤2 years	40 (18.6)	3 (18.8)	
>2 years or never	73 (34.0)	7 (43.8)	
HP infection status, *n* (%)			
HP-eradicated	125 (58.1)	9 (56.2)	1.00
HP-positive	90 (41.9)	7 (43.8)	

GC gastric cancer; *HP Helicobacter pylori*; *IQR* interquartile range.
